# The Influence of Kaolin Clay on the Mechanical Properties and Structure of Thermoplastic Starch Films

**DOI:** 10.3390/polym12010073

**Published:** 2020-01-02

**Authors:** Anita Kwaśniewska, Dariusz Chocyk, Grzegorz Gładyszewski, Jarosław Borc, Michał Świetlicki, Bożena Gładyszewska

**Affiliations:** 1 Department of Applied Physics, Lublin University of Technology, 20-618 Lublin, Poland; d.chocyk@pollub.pl (D.C.); g.gladyszewski@pollub.pl (G.G.); j.borc@pollub.pl (J.B.); m.swietlicki@pollub.pl (M.Ś.); 2 Department of Biophysics, University of Life Sciences, 20-950 Lublin, Poland; bozena.gladyszewska@up.lublin.pl

**Keywords:** biopolymer films, clay, nanocomposite, structure

## Abstract

The aim of study was to investigate the influence of kaolin on the physical properties and utility of film produced from native starch. The work involved measurements of strength, structure, and thermal properties. The films were prepared by the casting method. Composite films with 0%, 5%, 10%, and 15% kaolin additives were examined. Measurements of mechanical properties were carried out using the uniaxial tensile test, the nanoindentation test, and nanoscratching. Surface properties were examined by atomic force microscopy and contact angle measurements. Structure was determined by the X-ray diffraction method, and thermal properties were determined by differential scanning calorimetry. A significant influence of kaolin on the strength parameters and thermal and barrier properties of composite films was found. An increase in kaolin content reduced the tensile strength, Young’s modulus, and Poisson’s ratio. Structural analysis showed a partial intercalation and the layered arrangement of kaolin particles. Kaolin additives increased the barrier properties of water vapor in composite films of about 9%. Biopolymer modification by nanoclay reduced the thermal stability of composite films by 7% and could accelerate the biodegradation process. Increasing the concentration of kaolin in the biopolymer matrix led to heightened surface roughness (approximately 64%) and wettability of the surfaces of the film composites of 58%.

## 1. Introduction

One of the issues of great concern today relating to environmental protection is the increase in the production of packaging based on biodegradable materials [1]. Making biodegradable materials from cyclically renewable and environmentally friendly plant sources is creating opportunities to replace conventional petroleum products [2]. Such materials could be used as ecological disposable packaging, which could be composted with out-of-date or residual products [3].

Recent studies have shown that biopolymer materials usually have poor mechanical properties [4]. These materials are characterized by strong hydrophilic properties, which form a negligible barrier to water vapor [5]. Materials used for food packaging must create a suitable barrier to protect them from external factors to ensure their freshness and stability under various conditions of use [6]. In order to improve their mechanical properties and reduce the hydrophilic properties of starch films, it was proposed to introduce mineral nanofillers into the polymer matrix [7,8] instead of macrofillers such as cellulose fibers [9], cotton, jute, and bamboo [10]. Macro and micro additives used to modify polymers have usually uneven distribution in the polymer matrix. The result is local stress, and the film is not homogeneous and the boundary between the polymer and the additives are visible. Therefore, nanoadditives, which are characterized by good compatibility, uniformity dispersion, and strong connection with the polymer structure, have become functional fillers. One possible additive is a nanoclay that affects the mechanical, structural, and barrier properties of the composite films. The most common nanoclays are silicates and in particular layered silicates (phyllosilicates) such as montmorillonite, talc, and kaolin. Increasing the barrier properties is commonly associated with the layered arrangement of additive particles in the polymer matrix. [7,8,11,12,13].

Another issue is the use of different biopolymer plasticizers that affect film elongation, while significantly reducing its barrier properties [11]. Lower thermal stability causes faster degradation [14]. The addition of kaolin could increase the surface roughness [15].

The aim of this study was to examine the influence of unmodified kaolin on the properties of thermoplastic starch (TPS) films. Kaolin clay was used due to its non-toxicity, availability, and low cost. The films were prepared using the casting method. Four types of biopolymer film were prepared, all with 20% glycerol and 0%, 5%, 10%, and 15% kaolin content in relation to the starch dry mass, respectively. The mechanical, structural, thermal, and barrier properties were determined for the obtained films.

## 2. Materials and Methods

### 2.1. Materials

The raw material used to prepare the biopolymer films was potato starch produced by Melvit S.A. The native starch was a raw product, which was unmodified in any chemical, physical, or enzymatic way. The solvent in which the polymer solution was prepared was distilled water. Glycerol 99.5% produced by Avant Performance Materials was used as the plasticizer. Kaolin clay (Valentine Clays Ltd, Stoke-on-Trent, United Kingdom) was used as an extra additive. From diffraction measurements for kaolin powder, the average kaolin grain size was found to be 309 nm.

### 2.2. The Preparation of the Biopolymer Films

The biopolymer films were prepared by the casting method [4,16]. Kaolin suspension in 15 ml of distilled water was added to the prepared 105 ml aqueous solution of starch and plasticizer. To obtain a uniform suspension of kaolin, the mixture was treated with ultrasonic homogenizer for 180 seconds at 25 °C. This suspension was added to the polymer solution and treated with a magnetic stirrer rotating at 150 rpm and heated up to 80 °C. Then, the solution was mixed for 30 minutes. Next, the composite solution was poured into molds and kept in a climatic chamber until the solvent evaporated. Drying was carried out at 23 °C at 50% RH for 4 days. The films were conditioned in a desiccator. The material preparation method enabled homogeneous materials to be obtained under controlled atmospheric conditions, and resulted in the accurate determination of the fiber composition of the films. Samples were prepared with 20% glycerol and 0%, 5%, 10%, and 15% of added kaolin in relation to the starch dry matter, respectively. In the work, these samples are marked as k0, k5, k10, and k15.

### 2.3. Mechanical Properties

The Young’s modulus, Poisson’s ratio, tensile strength, and maximum strain were measured using the uniaxial tensile test. The measurements were carried out using the Deben Microtest (Deben Ltd. Suffolk, UK) with a 200 N maximum load. The samples were rectangular, with a width of 10 mm and a length of 20 mm. The initial distance between the grips was 10 mm, and the samples were elongated at a speed equal to 0.5 mm/min. Five independent tensile tests were performed for each kind of film.

An Ultra Nanoindentation Tester, with a Berkovich indenter (Anton Paar GmbH, Graz, Austria), was used for the hardness test [17,18]. Nanoindentation tests were performed with a depth of 5 μm at a rate of 10 mN/min. In order to avoid the risk of material creep under the influence of the indenter, a 10 s pause was applied in the test, when the indenter remained stationary in the material. A series of 10 indentations was made for each of the films. To prevent the influence of the substrate on the results, the depth of the indentation never exceeded 5% of the thickness of the sample.

The elastic recovery and friction coefficient were determined based on a scratch test carried out on a Nanoscratch Tester (NST) (Anton Paar GmbH, Graz, Austria) equipped with a spherical diamond indenter. The measurements were made with a linear progressive load from 2 to 100 mN, with a scratch length of 1.2 mm, at a rate of 0.12 mm/min. Five scratches were made for each film.

The thicknesses of the films were determined using an electronic micrometer QuantuMike (Mitutoyo, Kawasaki, Japan). The films were tested randomly, and the thicknesses were calculated by averaging the results of 20 replicate measurements.

### 2.4. Structure

The structural study was performed using a high-resolution Empyrean (Malvern PANalytical, Almelo, Netherlands) diffractometer equipped with a copper anode tube (λ = 0.15418 nm) operating at 40 kV and 30 mA, and equipped with a proportional detector. A 1/2° divergence slit and Soller slit for the incident beam path were used, while a 1/2° divergence slit and Ni filter were applied for the diffracted beam path. All measurements were carried out in θ–2θ geometry in the 2θ range from 10° up to 60°, with a step of 0.01°. The data were collected with the exposure time of 8 s.

### 2.5. Surface Properties

Surface topography was scanned in non-contact mode using an AFM MultiMode 8 (Bruker, Billerica, Massachusetts, USA) atomic force microscope. Each scan was performed at 1 Hz and 512 × 512 pixels at room temperature and 50% relative humidity. For each of the tested samples, images of the surface topography of three non-contiguous areas of 10 μm × 10 μm were collected. For each measurement, the roughness parameters were determined.

The wettability of the surface films was characterized by the static sessile drop method. The contact angle was determined based on the geometry of the water drop on the tested surface. The measurements were performed using an Attension Theta Little (Biolin Scientific, Espoo, Finland) optical goniometer. A drop of deionized water was deposited on the tested film using a microliter chromatography syringe equipped with a Type 3 chromatography needle (90°) with an internal diameter of 0.51 mm. Images of the drop geometry were recorded 5 seconds after the deposition on the surface. The measurement series consisted of 10 tests. The contact angle was measured on both sides of the drop, and the results were averaged.

### 2.6. Thermal Properties

The thermal properties of the films were tested using a DSC Mettler-Toledo DSC-1 STAR^e^ SYSTEM (Mettler-Toledo GmbH, Greifensee, Switzerland) differential scanning calorimeter, with heat flux flow measurement. The measurement was performed under a nitrogen gas atmosphere in the temperature range of 20 to 290 °C, and the scanning rate was 10 °C/min. For each sample, the measurement was repeated three times.

### 2.7. Water Vapor Permeability

To determine the water vapor permeability coefficient (WVP), the gravimetric method was used. It was based on measuring the loss of mass from the vessel over time under strictly defined conditions. The containers were filled with distilled water and sealed with Parafilm^®^M film. The surface area of the film, which the vapor could penetrate, was equal to a = 9.616 × 10^−4^ m^2^. The samples after preparation were weighed and kept in a climate chamber at 20 °C and 40% relative humidity. Three sets of weighing containers were prepared for each film, and the test was conducted for 7 days with daily weight measurements. A linear loss of mass relationship was identified based on the recorded results; then, the WVP (water vapor permeability) was calculated in accordance with Equation (2) in Ref. [19].

### 2.8. Statistical Analysis

Statistical analyses of the data obtained from the measurements were performed with the software package Statistica 13.1 (TIBCO Software Inc., Palo Alto, California, USA), using the one-way analysis of variance (ANOVA) and Tukey’s HSD test for significance level *p* < 0.05. The values given in the tables are expressed as an average standard deviation (±SD).

## 3. Results and Discussion

### 3.1. Mechanical Properties

To measure the mechanical parameters, the uniaxial tensile test was applied. In this test, sample strain versus tensile force was recorded [16]. Tensile strength σ_max_, elastic modulus E, and maximum strain ε_gr_, were obtained directly. The Poisson’s ratio was determined using the random marker method [20]. The method is based on image analysis of the sample surface onto which the graphite markers were randomly applied. In this method, we determine changes in the relative position of markers in the directions parallel and perpendicular to the loading axis. The advantage of the random mark method is that the results obtained are independent of the boundary conditions [21]. The determined parameters, together with their statistical analyses, are given in Table 1.

The presented results show a significant influence of kaolin on the mechanical parameters of the film. The tensile strength σ_max_ reached the highest value for the base film k0 at 6.22 MPa. The increase in kaolin concentration in the composite k15 resulted in a reduction of σ_max_ to 4.43 MPa. The value of the Young E modulus of composite film k15 was almost three times lower than that of the modulus of the base film. The Poisson’s ratio also reached the highest value for the film without the addition of a nanoclay, and was equal to 0.38, whereas the smallest value for the film with the highest amount of kaolin additive k15 was equal to 0.22.

The scratch test was used to measure the friction coefficient of the film and to calculate the surface elastic recovery [22]. The single scratch test consisted of three scans along the specimen surface. The obtained film surface topography before (P_f_) and after (R_d_) the scratch allowed to determine the surface elastic recovery (Table 1). Elasticity determines the ability to recover the original shape and dimensions after the load is removed. The calculated values of the elastic recovery and the coefficient of friction did not indicate any correlation between these parameters and the nanoclay content in the composite. 

**Figure 1:**
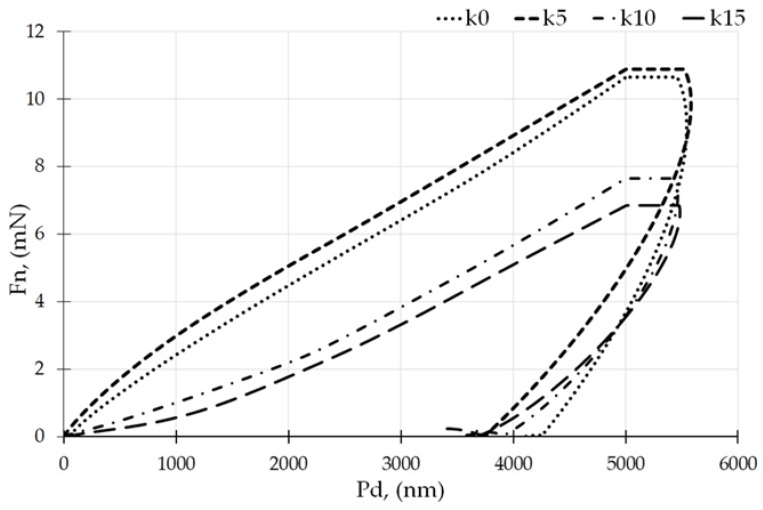
The representative curves of the force F_n_ vs. the indentation depth P_d_.

Examples of curves for the normal force F_n_ versus the indentation depth P_d_ from the nanoindentation test are shown in Figure 1. The obtained results were based on the Oliver–Pharr model and were calculated directly from the indenter force and the displacement curves [18]. Hardness H_IT_ decreased with an increase in the kaolin content in the composite matrix, from the highest for k0 equal to 19.1 MPa to the lowest for film k15 equal to 13.2 MPa.

### 3.2. The Structure of the TPS Films

**Figure 2:**
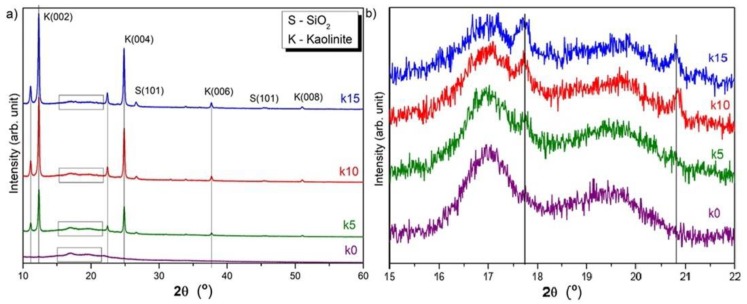
X-ray diffraction patterns of, (**a**) k0, k5, k10, k15 films; (**b**) an enlarged range of diffraction pattern for 2θ from 15° to 22°.

The X-ray diffraction profiles measured for the TPS film (sample k0), and the composite films (samples k5, k10, and k15) are presented in Figure 2. The X-ray diffraction profiles of sample k0 and the samples with additives are clearly different. High and very narrow peaks represent the crystalline structure of the additive. The analyses of the peak positions show that these peaks resulted from kaolin and silicon oxide.

Figure 2b presents an enlarged range for the diffraction pattern for 2θ from 15° to 22°. It appears from the profiles that the structure of the TPS matrix in all the tested films did not change. Wide peaks around the angle of 2θ = 17.1° and 19.8° can be identified as originating from B-type structures (2θ = 17.1°) [23] and from V_h_-type structures (2θ = 19.8°) [24,25]. 

The occurrence of B-type structures is associated with the recrystallization of amylose after production. In contrast, the occurrence of peaks derived from V_h_-type structures is commonly associated with the production process and the structures formed as a result of a reaction between the starch and glycerin. Müller et al. [26] and Jiang et al. [27] obtained a similar diffraction pattern for the starch film. The positions of the peaks corresponding to the crystal structure of the TPS were not altered, which means the lattice parameters were not affected by the amount of clay incorporation. These X-ray diffraction patterns indicated that the nanoclay did not influence the crystal structure of the TPS matrix in a significant manner.

The detailed phase analysis shows that the X-ray diffraction profiles of the films with the addition of kaolin reveal only peaks from the planes (002) of kaolin [28] and silicon oxide (101), and additional peaks around 2θ = 11° and 22°. The fact that the profiles of the samples with nanoclay revealed only (002) peaks indicates that the kaolin layers dispersed in the polymer matrix were layered and oriented with tetrahedrons toward the surface of the film. Similar properties were observed for the films tested by Lavorgna et al. [29], in which the layers of montmorillonite were also arranged parallel to the film surface. 

### 3.3. Surface Morphology

**Figure 3:**
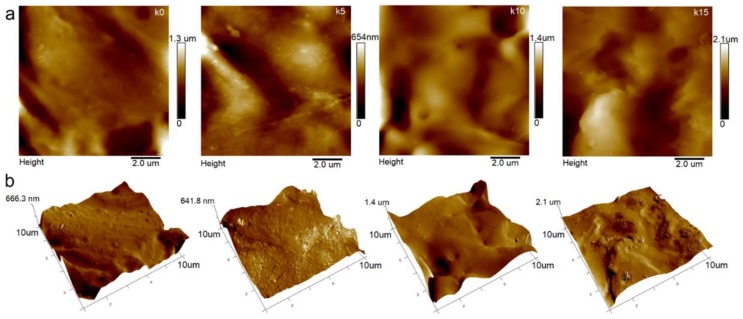
The surface texture of films with varying kaolin content as a height domain, measured using atomic force microscopy (AFM) (**a**) 2D; (**b**) 3D.

The 2D and 3D images of surface topography are presented as height domains in Figure 3. The images were corrected to eliminate the irregularities associated with the shapes of the samples. The root mean square heights (S_q_) and the arithmetical mean heights (S_a_) of the surfaces were determined by SPIPTM6.7.8 (Image Metrology), and the results are presented in Table 2. The surface roughness increased with an increase in the concentration of the nanoclay in the biopolymer matrix. The lowest S_a_ value was recorded for k0, and was 114.8 nm, and the highest for k15 was 187.7 nm. Gutierrez et al. [15] showed a similar increase in the roughness of the starch film S_q_ from 25 nm for pure TPS up to 80 nm for TPS with 4% MMT.

The wettability is described by the values of the contact angle as described in Section 2.5. The values of the contact angles are given in Table 2. The water contact angle θ_z_ is considered as an indicator of the surface’s hydrophilic or hydrophobic nature. In this case, the lowest contact angle of 38.5° was observed for the surface of the k15 film, indicating the highest wettability. However, the surface of the base film k0 showed hydrophobicity, reaching θ_z_ = 92.7°. Rekik et al. [30] presented the results of their studies on the wettability of chitosan film, depending on the percentage of added kaolin.

For the films without nanoclay, the contact angle value was 57° and was the smallest among all measured samples. This was opposed to the observed wettability of k0 film, for which this parameter reached the highest value among the samples tested. The addition of 1% of kaolin to the chitosan matrix caused an increase in the contact angle to 62°, while for 5% of nanoclay, the angle was 83°. The differences in the wetting of the chitosan film compared to the analyzed k0, k5, k10, and k15 most likely resulted from the different chemical structure of the chitosan bonds compared to the chemical structure of the starch.

### 3.4. Thermal Analysis

Differential scanning calorimetry was used to determine the temperature of melting and the temperature of degradation. All the samples were scanned in one heating cycle, and the results of the thermogram evaluation are presented in Table 3. The total area of the registered calorimetric peak corresponded to the enthalpy of the process during the transformation, which allowed to determine the phase transformation heat. 

With the increase in kaolin concentration, the melting temperature of the polymer decreased. The highest value was obtained for the base film k0, which is equal to 81.7 °C at ΔHM = −164.8 J/g. The lowest value was found for the k15 film with the enthalpy equal to −110.5 J/g. The temperature of the thermal degradation of the film decreased with the increase in the kaolin content. The highest T_D_ value was observed for the base film k0 (240.4 °C), and the lowest T_D_ was observed for the composite film k15, which is equal to 220.8 °C.

Lower values of melting temperatures for TPS starch films, in relation to our results, ranging from 54 to 74 °C, were shown by García et al. [31]. During our measurements, the film was loosely adjacent to the bottom of the crucible, so the film could deform while heated, and the thermal contact between the film and the measuring probe was weaker. These could explain the differences in the results obtained.

### 3.5. Barrier Properties

**Figure 4:**
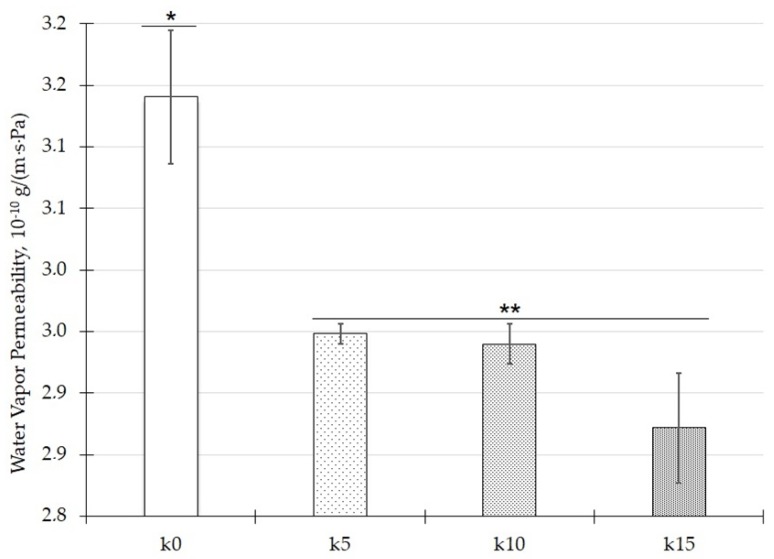
Water vapor permeability; data given are mean ± SD; (*, **) the marker means a group that is statistically significantly different (*p* < 0.05).

The water vapor permeability was defined as the amount of vapor passing through the films at any given time. This allowed to calculate the water vapor permeability (WVP) coefficient. The WVP values of the film are shown in Figure 4. It is clear that significant statistical differences (*p* < 0.05) existed between the barrier properties of the base film (*) and the composite films (**). The permeability value of the film k0 was equal to 3.14 × 10^−10^ g/(m·s·Pa), and it was the highest value of all the films. This corresponded to the weakest barrier. The lowest WVP was observed for the k15 sample, 2.87 × 10^−10^ g/(m·s·Pa). It can be concluded that the composite film had higher barrier properties than the base film k0. 

A similar relationship between WVP and the talc percentage content was observed by López et al. [13]. The WVP for the zero talc content film was 12.9 × 10^−10^ g/(m·s·Pa), which is the highest value of the tested samples. A 3% additive of talc decreased the WVP to 9.3 × 10^−10^ g/(m·s·Pa), while an increase in talc concentration to 5% only slightly changed WVP up to 9.6 × 10^−10^ g/(m·s·Pa).

### 3.6. Discussion

The addition of kaolin mainly improved the barrier properties of the TPS material. In the literature, increasing the barrier properties is commonly associated with the phenomenon of the layered arrangement of kaolin particles in the polymer matrix [29]. The orientation of the kaolin layers parallel to the surface of the film causes an increase in the water vapor barrier represented by the WVP coefficient. The vapor molecules have to overcome an impermeable silicate layer during the diffusion through the film that results in a longer path. This increases the time of vapor diffusion through the film and reduces the permeability. The layered arrangement of the kaolin particles in the matrix structure was confirmed by the diffraction measurements. In the X-ray diffraction profiles, only peaks from the planes (002) of kaolin and additional peaks around 2θ = 11° and 22° are present. On the other hand, the appearance of peaks for 2θ = 11° and 22° results from the kaolin layers, which incorporate with the polymer chains by means of hydrogen bonds. TPS biopolymer chains entered the interlayers of the kaolin particles. This might confirm the partial intercalation of the kaolin in the polymer matrix, as was also reported by Guarás et al. [32]. The intensity of the peaks (2θ = 11° and 22°) increases with the addition of the nanoclay. The increase in the X-ray diffraction peak intensity results from the larger amount of the parallel assembly of intercalated clay particles. On the other hand, the increase in the kaolin additions causes an increase in the percentage of polymer chains to be intercalated. The phenomenon of decreasing the concentration of the non-incorporated polymer chains might be a main factor influencing the mechanical properties of the composite films, as demonstrated particularly by the tensile strength and hardness measurements. Moreover, the results of the surface roughness (S_q_ and S_a_) and the contact angle θ_z_ measurements show the opposite correlation with the nanoclay additive content. If the addition of nanoclay increases, the wettability of the surface increases, too. The difference between the wettability of the tested films can be caused by surface irregularities associated with the presence of a filler in the composite. This was confirmed by an increase in the S_q_ and S_a_ roughness parameters with the kaolin additive content. Studies have shown that the decrease in transformation temperatures due to the biopolymer modification can affect the thermal stability of the polymer, accelerating its decomposition.

## 4. Conclusions

From the results presented here, the following conclusions can be drawn:

The physical modification of the TPS matrix with layered silica influences its mechanical properties. Compared to TPS biopolymer, composites with kaolin additive were characterized by lower strength properties.

Kaolin intercalated in the TPS matrix. The kaolin particles showed a layer arrangement in the structure of composite matrix and parallel to the surface of the film.

Kaolin does not change the crystal structure of the TPS matrix.

Kaolin increases the barrier properties for water vapor.

The addition of kaolin significantly increases the surface roughness of composite films and strongly influences their adhesive properties.

Biopolymer modification by nanoclay reduces the thermal stability of composite films and may accelerate the biodegradation process.

The improved barrier properties increase the suitability of composite films and suggest eventual applications as packaging material.

## Figures and Tables

**Table 1:** The mechanical parameters of the films.

	k0	k5	k10	k15
Thickness, (mm)	0.096 ± 0.03 ^a^	0.099 ± 0.04 ^ab^	0.102 ± 0.02 ^ab^	0.106 ± 0.01 ^b^
Tensile Strength, (MPa)	6.22 ± 0.35 ^a^	5.29 ± 0.29 ^a^	4.23 ± 0.36 ^b^	4.43 ± 0.32 ^b^
Young’s Modulus, (MPa)	98.39 ± 11.89 ^a^	55.18 ± 5.00 ^b^	38.14 ± 9.16 ^b^	37.25 ± 10.68 ^b^
Poisson’s Ratio, (–)	0.38 ± 0.06 ^a^	0.29 ± 0.04 ^b^	0.25 ± 0.03 ^b^	0.22 ± 0.03 ^b^
Maximum Strain, (–)	0.59 ± 0.06 ^a^	0.53 ± 0.09 ^a^	0.37 ± 0.03 ^b^	0.36 ± 0.02 ^b^
Hardness, (MPa)	19.1 ± 3.81 ^a^	17.9 ± 1.18 ^ab^	13.3 ± 2.53 ^bc^	13.2 ± 2.38 ^c^
Friction Coefficient, (–)	1.32 ± 0.02 ^a^	1.32 ± 0.05 ^a^	1.30 ± 0.03 ^a^	1.31 ± 0.03 ^a^
Elastic recovery, (%)	64	52	57	65

^a–c^ Superscripts in the same row mean statistically significantly different groups—Tukey’s HSD test (*p* < 0.05).

**Table 2:** The values of the roughness and contact angle parameters.

Film	S_a_, (nm)	S_q_, (nm)	Contact Angle, (°)
k0	114.8 ± 10.6 ^a^	130.5 ± 2.0 ^a^	92.69 ± 8.82 ^a^
k5	117.7 ± 25.4 ^a^	158.1 ± 1.9 ^ab^	48.03 ± 12.1 ^b^
k10	149.7 ± 19.8 ^ab^	194.5 ± 2.3 ^bc^	46.66 ± 11.58 ^b^
k15	187.7 ± 29.4 ^b^	242.9 ± 3.3 ^c^	38.46 ± 6.98 ^c^

^a–c^ superscripts in the same Column mean statistically significantly different groups—Tukey’s HSD test (*p* < 0.05).

**Table 3:** The calorimetric data.

Process	Melting		Thermal Degradation	
Sample	T_M_, (°C)	ΔH_M_, (J/g)	T_D_, (°C)	ΔH_D_, (J/g)
k0	81.7 ± 2.9 ^a^	−164.8 ± 4.0 ^a^	240.4 ± 2.4 ^a^	−130.1 ± 1.3 ^a^
k5	72.7 ± 0.2 ^b^	−106.4 ± 2.9 ^b^	226.2 ± 1.4 ^b^	−129.9 ± 0.9 ^a^
k10	75.4 ± 1.0 ^b^	−106.6 ± 3.1 ^b^	227.0 ± 0.3 ^b^	−124.0 ± 17.7 ^a^
k15	76.1 ± 0.5 ^b^	−110.5 ± 1.1 ^b^	220.8 ± 0.7 ^c^	−132.6 ± 2.9 ^a^

^a–c^ Superscripts in the same column mean statistically significantly different groups—Tukey’s HSD test (*p* < 0.05).
